# A Comprehensive Review on the Molecular Mechanism of Lycopene in Cancer Therapy

**DOI:** 10.1002/fsn3.70608

**Published:** 2025-07-13

**Authors:** Muhammad Maaz, Muhammad Tauseef Sultan, Muhammad Usman Khalid, Hassan Raza, Muhammad Imran, Muzzamal Hussain, Waleed Al Abdulmonem, Suliman A. Alsagaby, Mohamed A. Abdelgawad, Mohammed M. Ghoneim, Muhammad Asif Khan, Tadesse Fenta Yehuala, Samy Selim, Ehab M. Mostafa

**Affiliations:** ^1^ Faculty of Food Science and Nutrition Bahauddin Zakariya University Multan Pakistan; ^2^ Department of Food Science and Technology University of Narowal Narowal Pakistan; ^3^ Department of Food Science Government College University Faisalabad Faisalabad Pakistan; ^4^ Department of Pathology, College of Medicine Qassim University Buraidah Saudi Arabia; ^5^ Department of Medical Laboratory Sciences, College of Applied Medical Sciences Majmaah University AL‐Majmaah Saudi Arabia; ^6^ Department of Pharmaceutical Chemistry, College of Pharmacy Jouf University Aljouf Saudi Arabia; ^7^ Department of Pharmacy Practice, College of Pharmacy AlMaarefa University Riyadh Saudi Arabia; ^8^ Department of Human Nutrition and Dietetics University of Lahore Lahore Pakistan; ^9^ Faculty of Chemical and Food Engineering Bahir Dar Institute of Technology, Bahir Dar University Bahir Dar City Ethiopia; ^10^ Department of Clinical Laboratory Sciences, College of Applied Medical Sciences Jouf University Sakaka Saudi Arabia; ^11^ Department of Pharmacognosy, College of Pharmacy Jouf University Sakaka Saudi Arabia; ^12^ Pharmacognosy and Medicinal Plants Department, Faculty of Pharmacy (Boys) Al‐Azhar University Cairo Egypt

**Keywords:** anticancer, anti‐inflammatory, antioxidants, cancer cell modulation, DNA damage, lycopene, oxidative stress, PSA levels

## Abstract

The continuous progression of cancerous severity due to the interaction of physical (ultraviolet radiation), chemical (smoking, mycotoxins, and heavy metals), and biological (microbial populations) carcinogens inspired scientists to develop a correlation between active therapeutic agents and cancer proliferation. This review highlights the molecular mechanism by which lycopene imparts its anticancerous role, focusing on clinical and animal trials to validate its effectiveness. The antioxidant profile of lycopene promotes anticancerous properties that reduce cancer prevalence by activating cell signaling pathways and gene expression (involved in cancer cell proliferation). Lycopene's anti‐inflammatory properties suppresses the tumor growth and development‐promoting pathways, such as the PI3K/Akt/mTOR pathway. The anticancer property of lycopene is also evidenced by its inhibitory potential of the Wnt/β‐catenin signaling pathway that is involved in cancer cell modulation and propagation. Lycopene also suppresses and neutralizes oxidative stress and reactive oxygen species (ROS)‐induced DNA damage, preventing gene mutation. Inflammation‐induced cell death is mitigated by lycopene's anti‐inflammatory potential that lowers the expression of interleukin 6 (IL‐6) and tumor necrosis factor‐alpha (TNF‐α). Several clinical and randomized control trials have revealed the effect of lycopene supplementation in the management of cancer, including breast cancer, pancreatic cancer, prostate cancer, colon cancer, ovarian cancer, skin cancer, oral cancer, liver cancer, gastric cancer, and kidney cancer, by lessening prostate‐specific antigen (PSA) levels. These studies also accentuate certain limitations that need further trials to evaluate the long‐term consequences of lycopene supplementations and their specific effective dose.

## Graphical abstract

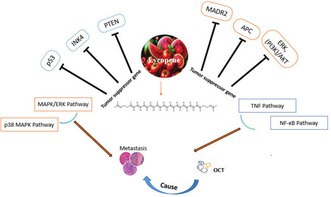

## 1 Introduction

Individual health is primarily affected by dietary habits, consumption patterns, environmental conditions, and lifestyle approaches. Food choices, ready‐to‐eat products, and reduced physical activity have elevated the disease burden worldwide (Oliveira et al. 2020). The prevailing conditions, including diabetes, hypertension, obesity, stroke, liver cirrhosis, and osteoporosis, attained special attention in functional food and nutraceutical industries. Various phytochemicals such as carotenoids, flavonoids, flavanols, saponins, anthocyanins, tannins, ellagitannins, phytoestrogens, phytosterols, polysaccharides, and catechins are isolated from the plant extracts that are used in nutraceutical as well as pharmaceutical industries to produce health‐promoting and disease‐mitigating drugs (Riar and Panesar 2024; Kamiloglu et al. 2022).

Lycopene, one of these phytochemicals and a red‐colored pigment, is extracted from different plant sources such as tomatoes, guava, watermelon, apricot, papaya, gac fruits, red grapes, oranges, mangoes, pomegranates, and carrots, and possesses numerous health benefits, that is, antioxidants, anti‐mutagenic, anti‐inflammatory, skin protective, cardioprotective, and neuron‐protective (Nieto et al. 2023). These properties increase its potential applications in the nutraceutical, pharmaceutical, food, and cosmetic industries (Ullah et al. 2020). Food industries utilize lycopene to manufacture colored and attractive food products. The colored attributes of lycopene are due to the long chromophores in the polyene chain that absorb significant frequencies of visible radiation (Caseiro et al. 2020). The review was structured by searching data from Google Scholar, PubMed, and Sci‐Hub to demonstrate the anticancer mechanism of lycopene. Data were collected by thoroughly reading research articles that inquired about the effect of various lycopene concentrations in inducing apoptosis and reducing the proliferation of cancer.

### 1.1 Lycopene's Bioavailability

Fruits and vegetables, particularly tomatoes, have traditionally been recognized as the major source of lycopene due to their high content of antioxidant compounds (Wu et al. 2024). In recent decades, modernization and industrialization have entirely changed the concept of food processing and development, which ultimately shifted consumers' trends toward the uptake of processed and ready‐to‐eat food products to obtain maximum lycopene contents (Vieira et al. 2021). Various studies have revealed multiple factors for modulating the bioavailability of lycopene. Baghabrishami and Goli (2023) revealed that the addition of tomato seed oil and heat treatment enhanced the bioavailability of lycopene. Similarly, digestible lipids, protein‐based emulsifiers, polysaccharide‐based texture modifiers (Liang et al. 2021), the addition of tomato pomace with excipient emulsions (Nemli et al. 2021), *Lactococcus lactis* and *Lactobacillus casei* fermentation (Kaur and Ghosh 2023), microencapsulation (Szabo et al. 2021), high‐pressure homogenization having 80 MPa pressure and 25°C temperature (Carpentieri et al. 2023), and freeze‐dried tomatoes (Tan et al. 2021) have improved the bioavailability and bioaccessibility of lycopene. Lycopene bioavailability is much better in processed foods than in fruits and vegetables. Pizza contains 32 mg/g wet weight of lycopene, while watermelon contains 4 mg/100 g wet weight of lycopene. Lycopene bioavailability is relatively limited in green tomatoes compared to ripened tomatoes due to the accumulation of carotenoids, volatiles, and organic acids (Arias et al. 2022). Several factors, such as environmental conditions, climatic fluctuations, and processing techniques, significantly affect lycopene absorption. Appropriate handling and heat treatment alongside fatty foods improves the bioavailability of lycopene in the human digestive tract (Jayarathna et al. 2023).

**FIGURE 1:**
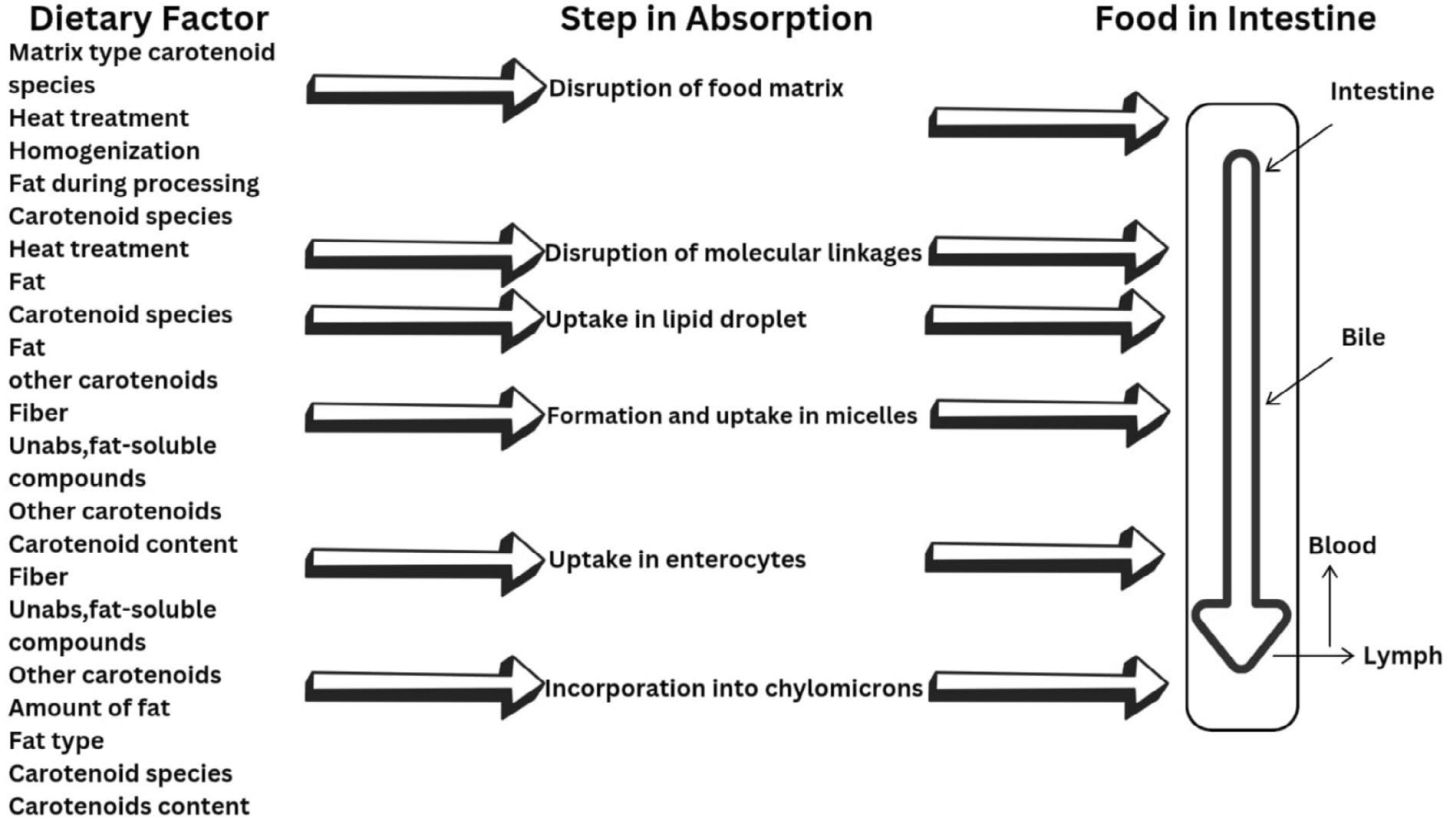
Carotenoid bioavailability & dietary factors affecting absorption.

Przybylska (2020) found that consuming fat‐rich salads bestowed higher lycopene bioavailability than the consumption of salad without any fat content. This isomerization occurs as lycopene reaches the food matrix in the stomach, which converts its Z‐isomers to the E‐state due to the thermal instability of Z‐isomers. The isomerized lycopene interacts with chyme to improve pH in the small intestine (duodenum) to assist the interaction of lycopene, fatty acids, and bile acids, eventually developing micelles that interact with enterocyte‐associated gut transporters such as Scavenger Receptor class B‐type I (SR‐B1), Niemann Pick C1‐like 1 (NPC1L1), and ATP‐binding cassette transporter (ABCA1). Afterward, they are incorporated into chylomicrons that enter through the lymphatic pathway into the bloodstream, forming LDL‐cholesterol and IDL‐cholesterol (Walther et al. 2019). The specific organs for lycopene storage are the liver, adrenal glands, testes, and adipose tissues, which are then utilized by the body on demand (Adetunji et al. 2021). Carotenoid bioavailability and dietary factors affecting absorption are presented in Figure 1.

### 1.2 Antioxidant Potential of Lycopene

Oxidative stress is a significant challenge for aggravating the risk of developing metabolic, cardiovascular, cerebrovascular, inflammatory, and arthritic disorders. Herbal plants constitute bioactive compounds such as naringenin, curcumin, ellagic acid, syringic acid, carotenoids, tannins, anthocyanins, and flavonoids that have beneficial effects in combating oxidative stress and oxidative disorders by scavenging reactive oxygen & reactive nitrogen species. Lycopene, the bioactive compound in tomatoes, rendered antioxidant potential by enhancing catalase (CAT), glutathione (GSH), superoxide dismutase (SOD), and mRNA antioxidant enzymes, as well as by suppressing the accumulation of amyloid beta (Aβ), amyloid precursor protein (APP), tumor necrosis factor‐alpha (TNF‐α), inducible nitric oxide synthase (iNOS), and β‐secretase (Imran et al. 2020). Lycopene's antioxidant profile was affirmed by administering 6.5, 15, and 30 mg/day of lycopene with low‐fat milk to healthy men and women (*n* = 18) with an age group > 40 years. Lycopene (30 mg/day) uptake for ~2 weeks significantly ameliorates lymphocyte DNA damage and urinary 8‐hydroxy‐20‐deoxyguanosine (8‐OHdG) (Bohn et al. 2021).

Lycopene also revealed antioxidant potential by reducing lymphocyte DNA damage when 12 mg/day of lycopene was consumed by healthy, non‐smoking postmenopausal women (*n* = 6) aged 50–70 years (Fenech et al. 2023). Approximately 10 mg/day of lycopene administration to diabetic individuals (*n* = 19) having specific age groups (35–70 years) for 2 weeks reduced malondialdehyde (MDA) to reveal its antioxidant potential (Leh and Lee 2022). Lycopene supplementation of 14 mg/kg for 4 weeks alleviated plasma TBARS (thiobarbituric acid reactive substance) and enhanced serum glutathione (GSH) concentrations in grade 1 hypertensives (*n* = 31) of 30–70‐year old individuals (Bin‐Jumah et al. 2022). The supplementing of 3 doses of lycopene capsules (15 mg/day) enhances lymphocyte count to manage oxidative stress among smokers (*n* = 13) and non‐smokers (*n* = 15) of 34.3 years average age (Hajizadeh‐Sharafabad et al. 2022). The evidence supporting the antioxidant potential of lycopene was provided by Gholami et al. (2021) when lycopene intake of 5–20 mg/day for 14 days reduced serum malondialdehyde (MDA) and elevated serum thiol levels in males (*n* = 6) and females (*n* = 6) of 31.3 years.

### 1.3 Experimental Studies

Lycopene imparts anticancerous potential by inducing apoptosis, modulating signal pathways, and improving enzymatic actions to reduce metastasis and invasion of tumor cells. One of the studies revealed the anti‐tumor potential of lycopene juice by enhancing serum lycopene and serum antioxidant levels and reducing oxidative stress‐stimulated DNA damage (Mirahmadi et al. 2020). Gloria et al. (2014) planned a human study to investigate the cancer‐mitigating effect of lycopene (40 mg/kg) that revealed a reduction in oxidative DNA damage, ultimately ameliorating cancer prognosis. However, lycopene supplementations alleviated androgen‐dependent and androgen‐independent CaP cell lines (involved in recombinant protein synthesis) and cancer cell proliferation (Kapała et al. 2022). Moreover, it has been observed that lycopene scavenges free radicals in normal and precancerous cells to activate the preventive mechanism; however, it modulates free radicals' production to aggravate oxidative stress in cancerous cells, which, in turn, promotes apoptosis induction, cell death, and DNA damage to cause cancerous cell death. These mechanisms promote the anticancer activity against PC_3_ prostate cancer cell lines (Ergul and Bakar‐Ates 2021), HT‐29 cells (Yulak et al. 2023), and Raji, K562, PC3, MCF‐7, and MDA‐MB‐231 cell lines (Ergul and Bakar‐Ates 2020).

**FIGURE 2:**
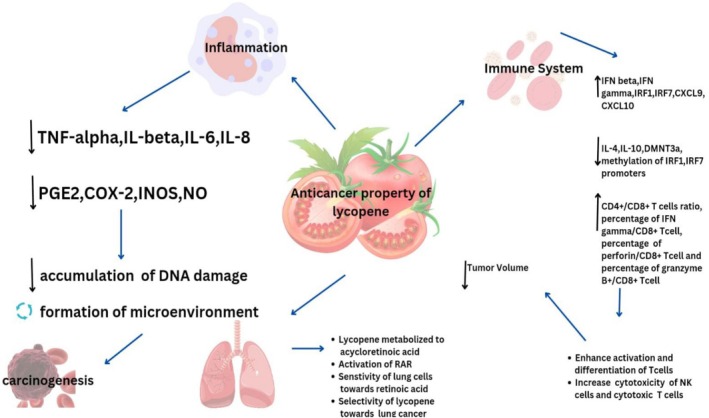
Anticancerous properties of lycopene.

Lycopene (30 mg/day), in addition to multivitamins and tomato sauce, has shown cancer‐reducing effects by reducing serum PSA (prostate‐specific antigen) concentration and by improving serum lycopene concentrations (Kapała et al. 2022; Laranjeira et al. 2022). Clinical trials presented the alleviation effect of lycopene (~30 mg/day) by reducing tumor size and modulating estrogen, androgen, and arachidonic acid metabolism (He et al. 2023; Issinger and Guerra 2021). Tumor development in LNCaP, PC3, and VeCaP cell lines was significantly reduced by 30 mg/day lycopene supplementation for 21 days (Khatoon et al. 2022). Lycopene supplementations (15 mg twice a day and 10 mg/day for 3 months) reduced cancer progression by lowering IL‐1, IL‐6, IL‐8, and TNF‐α levels (Khatoon et al. 2022; Koklesova, Liskova, Samec, Buhrmann, et al. 2020; Koklesova, Liskova, Samec, Zhai, et al. 2020). The administration of 20 and 50 μM lycopene arrested cell cycle and cancer cell proliferation; however, 10 μM of lycopene regulated the expression of PPAR‐γ, LXR‐α, and ABCA1 molecules (Kumar et al. 2021; Chi et al. 2021). Anticancerous properties of lycopene are shown in Figure 2.

### 1.4 Protective Molecular Mechanism of Lycopene

Cancer is a condition in which abnormal/cancerous cells proliferate to other parts of the body and become malignant. Cancer affects different organs of the body, such as the breast, prostate, pancreas, colon, mouth, esophagus, liver, kidney, brain, lungs, skin, cervix, and blood. Lycopene, an antioxidant, mitigates tumor cell propagation to normal cells of the body.

### 1.5 Breast Cancer and Lycopene

Breast cancer is a prevailing challenge among women in developed and developing countries, accounting for approximately 2.2 million cases worldwide in 2022. Lycopene is an antioxidative carotenoid that suppresses cancer growth and propagation. Shin et al. (2020) investigated the effect of lycopene & β‐carotenes on the cancerous cell lines (MCF‐7, MDA‐235, and MDA‐231) by treating them with 0.5–10 μM carotenoids for 48‐h and 96‐h intervals. Carotenoids revealed anticancerous effects by reducing cancer cell proliferation & cell cycle progression and promoting apoptosis after 96‐h intervals. The breast cancer‐mitigating effect of lycopene was investigated against SK‐BR‐3, MCF‐7, & MDA‐MB‐468 cancerous cells by lycopene supplementation for 7 days. The IC50 values manifested a dose‐ and time‐dependent response for MDA‐MB‐468, MCF‐7, and SK‐BR‐3 cell lines, and their respective values were 10 μM, 29 μM, and 22 μM. Lycopene revealed breast cancer protective effects by reducing cell cycle and tumor development (Islam et al. 2022). Puah et al. (2021) evaluated the inhibitory potential of various lycopene concentrations (0.1–20 μM) against H‐Ras MCF10A and MDA‐MB‐231 cell lines. It was concluded that the growth of H‐Ras MCF10A was inhibited by lycopene treatment for 24 h, with the IC50 value of 1.3 and 13 μM for MDA‐MB‐231 cancerous cells. Moreover, the anti‐proliferative effect of lycopene was also promoted by inducing apoptosis in H‐Ras MCF10A and MDA‐MB‐231 cancer cells.

**FIGURE 3:**
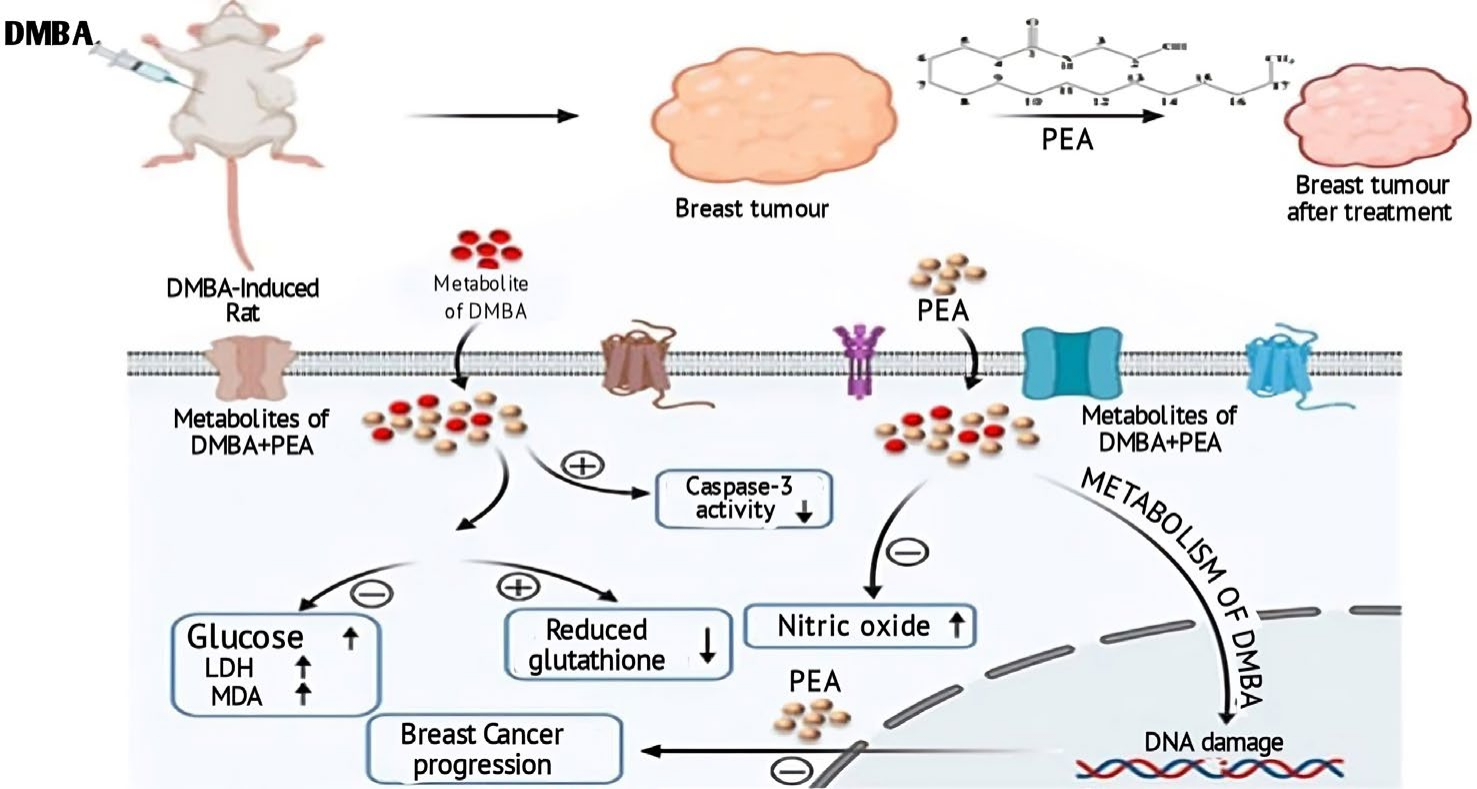
Breast cancer preventive mechanism of lycopene.

Lycopene portrayed time‐ and dose‐dependent breast cancer‐reducing effects by presenting the minimum inhibitory concentration (MIC) of 5 μM after 24‐h and 72‐h intervals (Nalewajska et al. 2020). Rowles III and Erdman Jr (2020) investigated the lycopene effect on breast cancer cells (MCF‐7) by conducting an MTT assay after 24‐h, 48‐h, and 72‐h intervals. The outcomes illustrated the dose‐ and time‐dependent mechanism of lycopene treatment that resulted in cell breakdown and shrinkage; however, their maximum results were achieved with higher doses at higher treatment intervals. The inhibitory effect of lycopene was determined against MCF‐7 breast cells by supplementing different concentrations (0, 2, 4, 6, 8, and 10 μM) of lycopene for 72 h. The MTT assay confirmed the tumor cell‐suppressing effect of lycopene by modifying Cytokeratin 8/18 (CK8/18) and CK19 protein expression in human breast cancer (MCF‐7) cell lines (Song et al. 2021). Furthermore, BRCA1 (Breast Cancer 1) and BRCA2, the tumor suppressor genes, are involved in strengthening genomic integrity by repairing DNA damage, modulating the transcription process, and arresting the cell cycle through the homologous recombination pathway. Moreover, these genes also aid in preventing oncogenesis by alleviating genetic mutations (Gorodetska et al. 2019). Black et al. (2020) conducted a study to evaluate lycopene's potential in modifying BRCA1 and BRCA2 gene expression in MCF‐7, HBL‐100, and MDA‐MB‐231 cancer cells. The fallouts exposed cell cycle arrest in the G1/S phase by 10 μM lycopene dosage for 24 h. Significant elevation of BRCA1 and BRCA2 expression was observed in MCF‐7 and HBL‐100 because of their stable genomic and functional DNA repair pathway capability, and the increased expression of BRCA1 and BRCA2 reveal the suppression of MCF‐7 and HBL‐100 cell lines proliferation (Chalabi et al. 2004). However, BRCA1 and BRCA2 expression were reduced in MDA‐MB‐231, as the MDA‐MB‐231 cell line is triple‐negative breast cancer (TNBC) cells and is highly invasive, with limited hormone receptors and a poor prognosis. The decreased BRCA1 and BRCA2 expression in this cell line are mainly due to its aggressive phenotype and defective DNA repair mechanism, which ultimately contribute to genomic instability, an important hallmark of cancer progression and therapeutic resistance (Katheeja et al. 2023). The tumor inhibitory effect of lycopene and genistein and their combination effect was studied against breast cancer by providing lycopene (20 mg/kg) and genistein (2 mg/kg) for 20 weeks. The results ameliorated tumor weight, adenocarcinoma masses, and breast cancer development that was associated with low levels of MDA, 8‐isoprostane, and 8‐OhdG (Rai et al. 2023; Kubczak et al. 2021). Figure 3 shows the breast cancer preventive mechanism of lycopene.

### 1.6 Pancreatic Cancer and Lycopene

Pancreatic cancer, the malignant form of cancer, is associated with aggravating levels of free radicals, oxidation‐induced stress, and inflammatory ailments within the digestive tract. The United States was among the pancreatic cancer countries, with an estimated 60,430 pancreatic cancer cases and 48,220 demises in the specific year 2022 (Zhao et al. 2024). Jeong et al. (2019) conducted a study to evaluate lycopene's potential in protecting against damage caused by pancreatic cells (PANC‐1 cells). It was found that ~0.25 μM or 0.5 μM lycopene minimized intracellular and mitochondrial reactive oxygen species (ROS) levels by inactivating NF‐kB and NF‐kB target genes (cIAP1, cIAP2, and survivin). The effect of lycopene on cerulein‐induced rats' pancreatic acinar cells was studied by injecting 2, 5, and 10 μmol/L lycopene for 72 h, significantly decreasing cytotoxicity and pancreatitis by lowering NF‐kB activity and arresting the JNK–caspase‐3 axis (Agah et al. 2021; Lee et al. 2021). Another study examined lycopene's protective effect against acute pancreatitis in 48 adult Wistar rats by supplementing 50 mg/kg lycopene for 10 days. Lycopene reduced the progression of L‐arginine‐induced acute pancreatitis by decreasing TNF‐β and enhancing glutathione (GSH) levels (El‐Ashmawy et al. 2018). A similar effect was observed in sodium taurocholate‐induced severe acute pancreatitis in rats when 10 mg/kg of lycopene diminished pancreatitis by slowing down NF‐kB p65 activity (Zhang et al. 2021).

The inhibitory effect of lycopene against pancreatic acinar cells was determined, it was found that IL‐6 and NF‐kB are suppressed by lycopene (2 or 5 μmol/L) after 2 h of cerulein activation, demonstrating their role in neutralizing ROS (Lee et al. 2022). Naviglio et al. (2019) explored the anticancerous potential of lycopene in protecting PANC‐1. They revealed pancreatitis reduction by lowering cell viability, cell death, and mRNA expression of survivin, cIAP1, and cIAP2 in affected cells. The severe acute pancreatitis lowering potential of lycopene was investigated in pancreatic acinar cells by administering lycopene for 24 h. It was observed that lycopene reduced severe acute pancreatitis by decreasing serum amylase, C‐reactive protein (CRP), TNF‐α, IL‐6, macrophage inflammatory protein‐1α, and monocyte chemotactic protein‐1. Moreover, myeloperoxidase (MPO), lipid peroxidase (LPO), and superoxide dismutase (SOD) levels showed significant reductions in ROS levels in pancreatic tissues (Yang et al. 2019). Lee et al. (2021) evidenced the inhibitory role of lycopene on ethanol/palmitoleic acid‐stimulated pancreatic acinar cells by suppressing NADPH oxidase‐mediated ROS production, IL‐6 expression, and zymogen modulation.

### 1.7 Prostate Cancer and Lycopene

Prostate cancer affects males in different regions, including Australian, Western European, and Northern American individuals. Approximately 1.4 million prostate cancer cases were reported, with a mortality rate of 26% (0.37 million deaths) in 2020 (James et al. 2024). Various studies have reported that lycopene exerts its prostate cancer suppressive effect by modulating cell cycle arrest at the G0/G1 phase, apoptosis induction by increasing the Bax/Bcl‐2 ratio, and down‐regulating androgen receptor expression. Moreover, lycopene also interacts with cancer cell‐suppressing pathways, such as PI3K, Akt, mTOR, MAPK, and EMT, to alleviate the proliferation of prostate cancer (Chen et al. 2022, 2014). Devlin et al. (2021) explored lycopene's effect in inhibiting prostate cancer progression among 40 individuals by administering 15 mg of lycopene a day continuously for 6 months. The results showed a significant decline in PSA and prostate cancer proliferation after 6 months of lycopene supplementation. A double‐blind, placebo‐controlled study was conducted among 105 African American men to investigate lycopene's impact on prostate cancer and benign prostate cancer development. The results revealed suppression of benign prostate cancer risk by lycopene supplementation (~30 mg/day) for 21 days (Ozkan et al. 2023). Mia et al. (2023) determined prostate cancer‐lowering properties of lycopene among 47,365 individuals. Its outcomes depicted that the consumption of lycopene and tomato products alleviated prostate cancer progression among consumers. Similar outcomes of lycopene on prostate cancer proliferation were observed by Matsushita et al. (2020) when elevated serum lycopene concentrations reduced prostate cancer prognosis.

The interaction between lycopene intake and prostate cancer development was explored by conducting a prospective study among 49,898 male individuals. An inverse correlation demonstrated a relative decrease in prostate cancer proliferation with higher dietary lycopene intake (Abir et al. 2023; Collins et al. 2022). Koklesova, Liskova, Samec, Buhrmann, et al. (2020); Koklesova, Liskova, Samec, Zhai, et al. (2020) and Katerji et al. (2019) also revealed similar results of dietary lycopene intake for prostate cancer development. Low prostate lycopene concentration was investigated in the pathogenesis of prostate cancer, and it was presented that lycopene ingestion (20–25 mg/day) for 6 months alleviated prostate cancer development (Mazurakova et al. 2022). Another study on 20 metastatic refractory prostate cancer patients was conducted to investigate the progressive role of lycopene on diseases. The results revealed that lycopene supplementation (10 mg/day) for 12 weeks lowered PSA levels among 30% of patients, consequently ameliorating prostate cancer severity, bone pain, and UTI (Maghsoudi et al. 2022). The unblinded, randomized, Phase I clinical study was designed by Mokbel et al. (2019) to investigate the impact of lycopene supplementation (30 mg/day for 4 months) on 81 Afro‐Caribbean prostatic intraepithelial neoplasia‐suffering men, and the results confirmed lycopene's prostate cancer inhibitory effect by lowering PSA levels. Randomized placebo‐controlled research was planned to determine lycopene's potential on DNA damage among 32 prostate cancer individuals. The results revealed significant alleviation in PSA concentrations, leukocyte DNA 8‐OH‐deoxyguanosine/deoxyguanosine (80HdG), and reduced DNA impairment by lycopene administration (~30 mg/day) for 3 weeks. However, the apoptotic process was significantly improved in hyperplastic and neoplastic cells with lycopene supplementation (Qi et al. 2021).

### 1.8 Colon Cancer and Lycopene

Colon cancer is ranked third among all types of cancers that affect the inner lining of the large intestine and is characterized by abdominal pain, cramping, diarrhea, constipation, and even bloody stools. Approximately 1.9 million colon cancer‐affected individuals were diagnosed throughout the world in 2020 (Merz et al. 2024). The inhibitory mechanism of lycopene against HT‐29 cells was investigated by providing different concentrations (0, 2, 5, and 10 μM) of lycopene for 3 days. Lycopene supplementations (~10 μM) for 3 days inhibited cancerous cell proliferation, the Akt signaling pathway, and non‐phosphorylated β‐catenin protein levels in HT‐29 cells (Ağagündüz et al. 2022). The anti‐proliferative mechanism of lycopene and eicosapentaenoic acid (EPA) on HT‐29 cells was studied to find the suppressive effect of lycopene and EPA on colon cancer cell propagation that was promoted by inhibiting the phosphatidylinositol 3‐kinase/Akt signaling pathway & mTOR molecule activation. Lycopene and EPA have synergistic effects on the up‐regulation of apoptotic proteins (Bax & Fas) (Kubczak et al. 2021). The colon cancer ameliorating effect of lycopene was studied in a mouse xenograft model that revealed the protective effect of lycopene for colorectal cancer by masking PCNA and β‐catenin protein expression in affected cells. Lycopene also exhibited its suppressive effect by reducing PGE2, COX‐2, and phosphorylated ERK1/2 protein (Marino et al. 2023).

Alhoshani et al. (2022) assessed lycopene and 5‐fluorouracil (5‐FU) potential against Caco2 by providing three μg/mL 5‐FU and 60, 90, and 120 μg/mL lycopene. Cytotoxicity, cell proliferation, and mitochondrial membrane potential of Caco2 colon cancer cells were reduced by the combination effect of lycopene and 5‐FU, while the Bax to Bcl‐2 ratio, caspase‐3, and caspase‐9 gene expression were improved. The anticancerous potential of lycopene and fish oil supplementation was revealed by the elevated expression of cell cycle inhibitors (p21 CIP1/WAF1 and p27Kip1) as well as by the reduced expression of MMP‐7, MMP‐9, COX‐2, PGE2, proliferating cell nuclear antigen, β‐catenin, cyclin D1, and c‐Myc protein (Islam et al. 2023). A randomized, placebo‐controlled study aimed to determine the effect of short‐term lycopene intervention on insulin‐like growth factor‐1 (IGF‐1)‐induced colon cancer. The outcomes depicted lycopene's anticancerous properties by elevating serum lycopene concentrations and reducing the IGF‐1‐binding protein‐3 M ratio (Langner et al. 2019). The combination effect of lycopene (2 & 5 mM), sulforaphane (2 & 5 mM), quercetin (25 mM), and curcumin (10 mM) was studied against colon cancer proliferation. It was observed that lycopene exhibited a chemoprotective role by suppressing mitochondrial activity, cancer cell propagation, cytotoxicity, and DNA synthesis (Zou et al. 2021). Lycopene inhibited colon cancer propagation by suppressing leptin‐mediated cell invasion, Akt activation, MMP‐7 expression, glycogen synthase kinase‐3β (GSK‐3β), ERK 1/2 proteins, AP‐1, and β‐catenin protein levels (Chang et al. 2020).

The combination effect of lycopene gold nanoparticles was studied in HT‐29 cell lines that found synergistic potential in reducing procaspases 8, 3, 9, PARP‐1, and Bcl‐2 expression, thereby inhibiting the proliferation of cancerous cells, the Akt pathway, and NF‐kB, MMP‐2, and MMP‐9 expression (Brar et al. 2021). Coelho et al. (2023) evaluated the anti‐inflammatory role of different lycopene concentrations (0, 10, 20, and 30 μM) on lipopolysaccharide‐induced SW 480 human colorectal cancer cells. The outcomes showed that lycopene supplementation restricted NF‐kB, TNF‐α, IL‐1β, IL‐6, iNOS, COX‐2, PGE2, and NO expression to demonstrate its protective role. The anticancerous effect of lycopene against HT‐29, T84, MCF‐7, A549, DU145, HepG2, Hela, and Hep‐2 was investigated by administering 1–5 μM of lycopene for 48 and 96 h. Lycopene alleviated cell growth in HT‐29, T84, and MCF‐7 after 48 h intervals. However, the apoptotic process was improved in T‐84, HT‐29, MCF‐7, and DU145 (Dai et al. 2021). Ataseven et al. (2023) revealed lycopene's (~8 μM) anticancerous effect in the HT‐29 cell line by improving caspase‐3, PARP, and 8‐oxy‐dG levels after 24 h of treatment.

### 1.9 Oral Cancer and Lycopene

Oral cancer affects oral cavities, lips, oropharynx, hypopharynx, and larynx, which account for an estimated 0.37 million cases worldwide in 2020. Specific pathways such as PI3K/AKT/mTOR, the Ras–Raf–MEK–ERK pathway, Wn signaling, the NF‐κB pathway, the Hippo pathway, and genes (P53, PTEN, CDKN2A, HRAS, PIK3CA, NOTCH1, IRF6, & TP63) are involved in oral squamous cell carcinoma (OSCC) pathogenesis (Kumari et al. 2024). Lycopene's anticancerous potential and its underlying mechanism were investigated against oral cancer occurrence by supplementing 0 μM, 0.5 μM, 1.0 μM, and 2 μM for 72 h. It was observed that lycopene treatment for 72 h repressed the proliferation, invasion, and migration of tumor cells as well as suppressing epithelial–mesenchymal transition by down‐streaming N‐cadherin, the PI3K/AKT/mTOR signaling pathway, p‐AKT, p‐PI3K, p‐mTOR, and bcl‐2 levels (Wang et al. 2020). The anticancerous potential of lycopene and β‐carotenoids was assessed in human oral cancer cells (KB‐1 cells). The results depicted that ~3 and 7 μmol/L of lycopene and 3 μmol/L of carotenoids significantly improved gap‐junction communication by elevating connexin‐43 expression in KB‐1 cancer cells (Abir et al. 2023; Ram et al. 2020). Lycopene lowered the risk of oral leukoplakia among 58 patients with 4 and 8 mg/day lycopene for 12 weeks. The results showed significant improvement (66% & 80%) in human oral leukoplakia treatment (Kamran et al. 2022).

Girisa et al. (2021) evaluated the combined potential of lycopene and curcumin against OSCC. Curcumin and lycopene supplementation for 72 h inhibited cancer cell viability and proliferation; however, the apoptosis process was improved in oral squamous cell carcinoma cells. Tao et al. (2021) designed a similar study to inquire about lycopene's inhibitory effect on OSCC cells by administering different concentrations (0.25, 0.5, 1, and 2 μM) of lycopene for 12 to 72 h. Lycopene intake reduced squamous cell carcinoma by inhibiting cell proliferation, cell migration, tumor growth, IGF1, IGF (BP), IGFBP3, and transcription factor Jun/Ap‐1. A randomized control trial (RCT) was conducted to differentiate the potential of lycopene gel and *Calendula officinalis* gel in 30 oral cancer patients by supplementing 2 mg lycopene and 2 mg *Calendula officinalis* gel. It was found that supplementation of lycopene gel and *Calendula officinalis* gel for 12 weeks resulted in lesion size suppression (Sahoo et al. 2022). Consuming approximately 8 mg of lycopene capsules daily for 2 months improved the treatment response and relief of burning sensations when Al‐Maweri et al. (2023) planned an RCT among 15 oral lichen planus patients. Sandhu et al. (2022), Rodríguez‐Molinero et al. (2021), Rao et al. (2020), and Gupta et al. (2020) also observed the same results of lycopene to manage OSCC patients.

Eita et al. (2021) conducted an RCT among erosive oral lichen planus individuals to determine lycopene's inhibitory mechanism by administering a 10 mg lycopene dose for two consecutive months. It was found that lycopene consumption for 2 months resulted in the down‐regulation of 8‐isoprostane, pain severity, and lesion activation. Pain, oral mucositis, and salivary MDA were significantly decreased in oral lichen planus sufferers when Hazzaa et al. (2021) administered 10 mg of lycopene capsules to the oral lichen planus individuals for 2 months. A comparative study investigated lycopene and spirulina capsules' capability to inhibit the proliferation of oral submucous fibrosis by providing lycopene capsules (8 mg) and spirulina capsules (500 mg) for 3 months. The results depicted that lycopene capsules were more efficacious in improving mouth opening and reducing burning sensations than spirulina capsules (Gopinath et al. 2022). A research trial compared lycopene and triamcinolone's potential in managing oral submucous fibrosis by supplementing lycopene capsules (6 mg/day) and triamcinolone injection (40 mg/mL) for 2 months. The applied treatment revealed that lycopene supplementation had better burn sensation relieving potential than triamcinolone injection (Roy et al. 2021). The comparative study was evaluated to determine the effect of lycopene and *Curcuma longa* against oral submucous fibrosis. Better results were obtained from 8 mg lycopene capsules as compared to 800 mg *Curcuma longa* in improving oral opening and reducing fibrous bands among oral submucous fibrosis patients (More et al. 2020).

Burning sensation, mouth opening, swallowing, and speech ability improved after 3 months of consuming 8 mg lycopene and oxitard capsules (Saso et al. 2022). Lycopene supplementation (8 mg) for 9 months resulted in mouth opening, tongue protrusion, check flexibility, and burning sensation relief improvement (Sarode et al. 2021). Tp et al. (2019) conducted an RCT in 40 oral submucous fibrosis patients by treating them with 2 mg lycopene capsules (twice a day) and injection of local anesthesia (0.5 mL) and hyaluronidase (1500 IU twice a week) for 3 months. The outcomes revealed equal contributions in modulating oral cancer and reducing burning sensations. Similarly, Arakeri et al. (2020) and Johny et al. (2019) presented significant improvement in mouth opening in grade II oral submucous fibrosis patients with 8 to 10 mg lycopene for 6 months. Manas et al. (2022) demonstrated that lycopene (3 mg), vitamin E (200 IU), and selenium (100 mcg) contributed to the management of oral leukoplakia.

### 1.10 Liver Cancer and Lycopene

Hepatocellular carcinoma (HCC) is a global prevailing condition that accounts for an estimated 70% to 85% of cases (Danpanichkul et al. 2024). Lycopene has a significant effect in lessening liver cancer development and proliferation. Lycopene supplementation (20 mg/kg) reduced the risk of high‐fat diet (HFD)‐induced non‐alcoholic fatty liver disease (NAFLD) by reducing body weight, serum LDL levels, total cholesterol, and improved the enzymatic potential of SOD and GPX (Kulawik et al. 2023). Róvero Costa et al. (2019) observed that lycopene (10 mg/kg) reduced NAFLD prevalence by minimizing LDL‐cholesterol, total cholesterol, and triglycerides, as well as by elevating high‐density lipoprotein‐cholesterol levels in high‐caloric diet‐induced obese rats. Reduced TNF‐α and MDA levels reflected the diminishing inflammation and oxidative injury in rats. The NAFLD in mice caused by consuming a high‐cholesterol and high‐fat diet was mitigated by 10 mg/kg lycopene. Lycopene reduces NAFLD by reducing CD4 & CD8 T cells, IFN‐γ, TNF‐α, macrophages, liver inflammation, and oxidative stress (Ni et al. 2020). Lycopene (10 mg/kg) promoted PPAR‐α and PPAR‐γ in high‐fat diet‐consuming mice, consequently lowering the severity of NAFLD (Yao and Liu 2022). Zhu et al. (2020) evaluated lycopene effectiveness in HFD‐fed mice suffering from NAFLD. The outcomes showed lycopene's potential in mitigating NAFLD by regulating SIRT1/AMPK and NAMPT and by reducing NF‐kB, IL‐6, IL‐1β, and TNF‐α activation. A cohort study planned to explore lycopene extract potential in managing NAFLD, alcoholic fatty liver, and fatty liver disease. Lycopene consumption was predominant in mitigating the severity of liver disorders by inhibiting NF‐kB and cholesterol levels (Donghia et al. 2024). Lycopene consumption (0.625–5.0 μM) improved PPAR‐α, retinoid X receptors‐β, retinoid X receptors‐γ, and potential and lowered HSC stimulation that revealed lycopene's capability in preventing liver fibrosis (de Barros Elias et al. 2019). Huang et al. (2022) investigated lycopene's impact in ameliorating liver fibrosis in bile duct‐ligated rats. It was observed that approximately 20 mg/kg improved eNOS (endothelial nitric oxide synthase), Akt (protein kinase B), and PI3K (phosphatidylinositol 3‐kinases) expression in hepatic endothelial cells.

Lycopene lowered the severity of hepatic ischemia–reperfusion injury by elevating hepatic cells' viability and down‐regulating IL‐6, TNF‐α, and MDA levels in AML12 cells (Liu et al. 2020). Xue et al. (2021) concluded that lycopene (20 mg/kg) supplementation minimized IL‐1β, TNF‐α, and IL‐6 levels by reducing macrophages and neutrophils' accumulation, consequently limiting the pathogenesis of ischemia–reperfusion injury in mice. Lycopene intake (~5 mg/kg) reveals its potential in mitigating hepatocellular cancer (HCC) by reducing mitochondrial anomalies, micronucleated cell scores, reactive oxygen species, lipid peroxide levels, liver enzymes, tumor proliferation, cyclin D1, PCNA, hexokinase, aldose, phosphoglucoisomerase, and glucose‐6‐phosphate (Ibrahim et al. 2021; You et al. 2021). Man et al. (2021) inquired about lycopene's potential in managing steatohepatitis and hepatocellular carcinoma (HCC) development. Lycopene supplementation (2.2 mg/kg/6.6 mg/kg) aided in managing steatohepatitis and HCC by attenuating body weight, liver weight, hepatic lesions, cyclin D1 levels, NF‐kB, and MMP‐2. Laranjeira et al. (2022) evaluated lycopene's potential in mitigating HFD‐induced HCC proliferation, and it was revealed that approximately 100 mg/kg of lycopene intake for 24 h enhanced hepatic lycopene storage and inhibited HFD‐induced carcinoma by suppressing β‐catenin protein, Met mRNA, & mTOR complex‐1 activation. The proliferation and growth of the K‐Hep1 human hepatoma cell line was suppressed by lycopene supplementation of 110 μM that downregulated cell growth, cell count, and cell invasion (Falsafi et al. 2022). Mekuria et al. (2020) evaluated the antimetastatic potential of lycopene (0.1–5 μM) on SK‐Hep‐1 cells that inhibited NADPH oxidase 4 (NOX4) expression and ROS levels that depicted the antimetastatic potential of lycopene.

### 1.11 Gastric Cancer and Lycopene

Gastric cancer is the leading cause of death in developed and developing countries and is characterized by the presence of *Helicobacter pylori* in food products (Pan et al. 2024). The gastric cancer‐lowering mechanism of lycopene was studied on GES‐1 and AGS, SGC‐7901, and Hs746T cell lines by providing different lycopene concentrations (0, 2.5, 5, and 7.5, 10 μM). Lycopene supplementation suppressed mitochondrial membrane potential by arresting the cell cycle. Lycopene intake stimulated the apoptosis mechanism and modified the expression of CCNE1, TP53, and the MAPK pathway (Zhou et al. 2023). Lycopene suppressed oxidative DNA damage by reducing HIF‐1α, NDRG1, Cyr61, STAT2 mRNA, and BNIP3, as well as by up‐regulating antioxidant enzyme activities (Cui et al. 2020; Jampilek et al. 2019). Lim and Wang (2020) evaluated lycopene's potential in lowering gastric cancer progression. It was concluded that lycopene mitigated gastric cancer proliferation by modulating inflammatory markers (increasing IL‐4 & IL‐10 and decreasing IL‐6 & TNF‐α), inducing apoptosis (elevating levels of caspase‐3 & Bax1), and arresting the cell cycle (reducing cyclin 1 & PCNA).

The lycopene effect was evaluated against N‐methyl‐N′‐nitro‐Nnitrosoguanidine (MNNG)‐induced gastric cancer using male Wistar rats by administering 50–150 mg/kg/day lycopene. The results revealed that lycopene improved SOD, CAT, GPx, IL‐2, IL‐4, IL‐10, TNF‐α, IgA, IgG, and IgM levels (Khalil et al. 2021). Lycopene supplementation of 0.125 mg/mL and 2.5 mg/kg for ~5 months elevated GSH, GPx, GST, and GR levels in MNNG and S‐NaCl‐induced gastric carcinogenesis (Gunes‐Bayir et al. 2022; Ilic and Ilic 2022). A cross‐control study was conducted among 80 stomach cancer individuals to inquire about the potential of dietary lycopene and cryptoxanthin in managing cancer incidences. Outcomes depicted an inverse correlation between lycopene and gastric carcinoma, exhibiting a strong anticancerous effect of lycopene (Sengngam et al. 2022). Gastric juice, SOD, MDA, MMP‐9, and MCP‐1 levels were significantly reduced by consuming lycopene, which revealed lycopene's potential in managing the severity of ethanol‐induced acute cancer injury (Chen et al. 2021). Lycopene depicted a gastric cancer‐mitigating effect by reducing ROS, β‐catenin stimulation, and nuclear translocation, and inducing apoptosis (Kim et al. 2019). Zhao et al. (2023) explored the lycopene inhibitory effect on gastric cancer cell lines by providing lycopene for 3 days. Lycopene restricted cancer cell line proliferation by elevating LC3‐1 and p‐ERK protein expression. Lycopene anti‐proliferative potential was studied against AGS cells by supplementing different lycopene concentrations (0.5, 1, and 2 μM) for 24 and 48 h. Lycopene stimulated apoptosis, DNA fragmentation, reduced cell viability, ROS, MAPK signaling pathway, NF‐kB modulation, and COX‐2 expression to overcome gastric cancer (Han et al. 2019). Lycopene supplementation reduced cell proliferation and cell growth via arresting the cell cycle, β‐catenin, deactivating Janus‐activator kinase 1/signal transducers, and *H. pylori‐induced* hyperproliferation (Park et al. 2019).

### 1.12 Kidney Cancer and Lycopene

Lycopene exhibited substantial effects in the management of renal disorders such as oxidative stress/cisplatin‐induced nephropathy, chronic kidney disease (CKD), renal carcinoma, and diabetic nephropathy. Several studies have revealed that lycopene hinders the potential of cancerous cells. Guo et al. (2021) conducted a study to investigate lycopene's potential in preventing the pathogenesis of cisplatin‐induced nephropathy in male Wistar rats (*n* = 28). It was observed that lycopene administration (6 mg/kg) reduced body weight, serum creatinine and urea levels, MDA, 8‐isoprostane, and renal Bax protein levels. An Eker rat model was employed to determine the effect of lycopene on the proliferation and development of renal cell carcinoma. Specific concentrations (0, 100, and 200 mg/kg) of lycopene were supplemented to 90 ten‐week‐old female Eker rats for 18 months. The results revealed that tumor growth, size, length, and quantity were significantly reduced, demonstrating the alleviation of renal cell carcinoma (Jurić et al. 2022). The effect of lycopene was assessed against contrast‐induced nephropathy by administering 4 mg/kg lycopene to the affected rats for 10 days. The results portrayed an increase in urea, creatinine, and malondialdehyde (MDA) levels, while glutathione (GSH), superoxide dismutase (SOD), catalase (CAT), and glutathione peroxide levels were significantly decreased. Lycopene lowered inflammatory, autophagy, and apoptotic mechanisms to ameliorate contrast‐induced nephropathy proliferation (Tsigalou et al. 2020). Lycopene down‐regulates the invasion, metastasis, and proliferation of cancerous cells by modulating the autophagy pathway. It is the most emerging pathway, which damages the defective organelles, proteins, and ROS to maintain genomic integrity. Lycopene has the potential to activate autophagic pathways in normal or precancerous cells by stimulating autophagy‐related markers, that is, LC3‐II and Beclin‐1, alongside reduced p62 levels (Tang et al. 2008). It has also been observed that lycopene aids in managing cervical cancer by elevating apoptotic (caspase‐3, PARP, and Bax) and autophagic (LC3β) expression (Parlak et al. 2024). Moreover, El‐Masry et al. (2024) also revealed similar results when lycopene and sorafenib were supplemented to alleviate metastasis in MDA‐MB‐231 cell lines. A double‐blind, randomized controlled research study was planned to evaluate lycopene's inhibitory role against cisplatin‐induced nephropathy among 120 patients, and the effect was achieved by attenuating glomerular filtration rate (GFR) and blood urea nitrogen (BUN) levels (Mahmoodnia et al. 2017).

Albrahim and Robert (2022) evaluated the potential of lycopene to manage high‐fat diet‐induced metabolic syndrome and kidney disease by supplementing 25 and 50 mg/kg/day of lycopene continuously for 3 months. Lycopene administration significantly ameliorated body weight, glucose, urea, creatinine, LPO, protein carbonyl (PC), TNF‐α, IL‐1β, and Bax levels. However, Nrf2 mRNA expression and enzymatic potential of SOD, CAT, GSH, and GPx were relatively elevated by lycopene supplementation. Tan et al. (2023) investigated the potential of lycopene on Adriamycin‐induced heart and kidney deadlines by providing 4 mg/kg lycopene for 3 days. The results depicted limitations in MDA, GSH, and CAT levels with lycopene supplementation. Selenium nanoparticles prepared from lycopene significantly minimized kidney weight, neutrophil gelatinase‐associated lipocal, creatine & urea levels, and IL‐1β, TNF‐α, & IL‐6 levels, while SOD, GSH, MDA, Nfe2l2, NO, and Hmox‐1 expression were improved (Al‐Brakati et al. 2021). Lycopene activated the Nrf2 signaling pathway by improving the expression of Bcl‐2 and decreasing NF‐kB, TNF‐α, caspase‐3, caspase‐9, and Bax expression. These properties of lycopene reduced inflammation and severity of aristolochic acid‐induced nephropathy (Wang et al. 2022). Lycopene's potential was determined by Deng et al. (2020) against cisplatin‐induced acute renal injured rats by administering 6 mg/kg of lycopene for ~2 weeks. The results showed that serum urea nitrogen, creatinine, and kidney efflux transporters MRP2 and MRP4 proteins were minimized, representing a preventive role in mitigating acute renal injury.

The growth and proliferation of renal cancerous cells A549 and ACHN were suppressed by lycopene supplementation of 2.5 μL (Pogoda et al. 2020). Tao et al. (2018) evaluated lycopene's suppressive mechanism against ochratoxin‐A‐induced oxidative stress in the kidney with 5 mg/kg of lycopene for 15 days. There was a significant reduction in SOD, GSH, catalase, and malondialdehyde (MDA) levels, revealing their effect in improving renal oxidative stress. Lycopene (5 mg/kg) supplementation for 4 weeks mitigated di‐ethylhexyl phthalate (DEHP)‐stimulated renal cell damage by reducing uric acid, creatinine, and blood urea nitrogen levels, and by suppressing kidney injury molecule‐1 (Kim‐1), cytochrome P450, and aromatic hydrocarbon receptor (AhR) nuclear transporter (Li et al. 2021). Lycopene extracted from *Lycopersicum esculentum* reduced doxorubicin‐induced nephrotoxicity by down‐regulating serum creatinine, urea, and lipid peroxidation levels and by elevating glutathione peroxidase, glutathione reductase, and catalase potential (Elfadadny et al. 2023).

### 1.13 Brain Cancer and Lycopene

Lycopene revealed its preventive brain cancer mechanism by ameliorating IL‐8, IL‐6, and IL‐1, deactivating the NF‐kB signaling pathway, and activating brain‐derived neurotrophic factors (Saini et al. 2020). A randomized placebo pilot study evaluated lycopene's potential on high‐grade gliomas by supplementing an 8 mg daily dose of lycopene to 50 carcinoma patients. Lycopene supplementation reduced the progression of high‐grade gliomas by improving the efficiency of radiotherapy (Qi et al. 2022). Lycopene's protective effect on hyperlipidemia‐stimulated brain damage was evaluated by administering different doses (5, 25, 45, 65, and 85 mg/kg/day) of lycopene for 4 weeks. Lycopene (25 mg to 85 mg) significantly reduced TC, triglycerides (TG), LDL‐cholesterol, oxidized low‐density lipoprotein (ox‐LDL), IL‐1, and TNF‐α levels in the brain and blood. However, LDLR receptors, nerve growth factor (NGF), gamma‐aminobutyric acid (GABA), and 5‐hydroxytryptamine (5‐HT) levels were increased in the brain hippocampus (Yang et al. 2018). Lycopene revealed a dose–response relationship against hyperlipidemia‐induced neuronal damage by reducing TC, TG, and LDL‐cholesterol and by upgrading claudin‐5 and neurons in the hippocampal region of the brain (Wang et al. 2018).

The therapeutic effect of lycopene was evaluated in managing middle cerebral artery (MCA)‐promoted ischemia/reperfusion brain injury in rats by providing 1, 2, and 5 μM of lycopene. Lycopene suppressed lipid peroxidation in brain homogenates and nitrite production in microglia and reduced infarct size in ischemia/reperfusion brain injury (Jin et al. 2021). Lycopene supplementation (40 mg/kg) attenuated the burden of early brain injury (EBI) followed by subarachnoid hemorrhage (SAH) through diminishing brain edema, blood–brain barrier (BBB) malfunctioning, and neuronal deficiencies (Brandes and Gray 2020). The lipophilic nature of lycopene permits it to cross the BBB through passive diffusion to induce a neuroprotective effect against neurological ailments (Guo et al. 2019). A study conducted by Ko et al. (2024) demonstrated that lycopene aids in managing glioblastoma multiforme by penetrating BBB, apoptosis induction, and cell cycle regulation. Farouk et al. (2021) and Setianingrum et al. (2020) investigated the protective and regulatory effect of lycopene against acrylamide‐induced neurotoxicity (causing brain dysfunction). Lycopene administration (10 mg/kg/day) for 21 days to 40 male albino rats elevated glutathione (GSH), glutathione peroxidase (GPx), malondialdehyde (MDA), nitric oxide (NO), protein carbonyl (PC), and neurotransmitters (dopamine, serotonin, & acetylcholinesterase) levels, demonstrating an efficacious role in mitigating neurotoxicity on rat brain cells. Lycopene dissolved in olive oil injected for 3 weeks showed its protective role in alleviating β‐amyloid protein (Aβ)‐induced Alzheimer's disease by reducing MDA levels and by enhancing antioxidant potencies to down‐regulate astrocytosis and microgliosis (Guo et al. 2023; Huang et al. 2019).

### 1.14 Cervical Cancer and Lycopene

Cervical cancer is the fourth leading cancer‐associated death that is caused by human papillomavirus (HPV) infection, E3 ubiquitin ligase involvement, oncogene activation (HCCR‐1 & HCCR‐2), and tumor suppressor gene inactivation (Wang et al. 2024). The newly diagnosed cervical cancer incidences are estimated to be ~11,500 per year in the United States (US), out of which 4000 women have a compromised survival rate. The reduced serum antioxidant levels (β‐carotenes, lycopene, canthaxanthin, retinol, and α‐tocopherol) are involved in the occurrence of cervical cancer (Letafati et al. 2023). Lycopene is a potent antioxidant that has a protective role in the occurrence of cervical cancer. Aktepe et al. (2021) investigated lycopene's anticancerous mechanism in Hela by providing 1 μM cisplatin, 10 μM lycopene, and their combination for 72 h. It was observed that lycopene and cisplatin act synergistically to arrest cell growth by enhancing Bax & Nrf2 expression and inactivating Bcl‐2 and NF‐kB pathways. Lycopene revealed its protective role in cervical intraepithelial neoplasia by improving serum lycopene concentrations among 32 Black women (Ferrari et al. 2023). The potential of lycopene's silver (AgNPs), iron (FeNPs), and gold nanoparticles (AuNPs) was determined against cervical cancer, and it was found that lycopene silver nanoparticles (LyAgNP) suppressed the progression of HeLa cells and COLO320DM cells (Shejawal et al. 2021; Yadav et al. 2020). Hajiesmaeil et al. (2022) found that a high intake of fruits and vegetables enriched with antioxidants and polyphenols has a significant influence in reducing the prevalence of cervical intraepithelial neoplasia. According to Ono et al. (2020), fat‐soluble vitamins, polyphenols, carotenoids, lycopene, cryptoxanthin, and folate inhibited HPV to reduce cervical cancer proliferation. Woźniak et al. (2021) portrayed an inverse correlation between serum carotenoid, retinol, and tocopherol concentrations and cervical cancer proliferation.

### 1.15 Uterine Cancer and Lycopene

Uterine cancer is a prevailing condition among females that has an incidence rate of 8.7 per 100,000 individuals. Adenocarcinoma and sarcoma are the major types of uterine cancer that have adverse effects on the reproducibility and fertility of women (Jawa et al. 2024). Lycopene demonstrated its protective effect against tumor development by suppressing cancerous cells' progression and proliferation. The anti‐tumor potential of tomato powder supplementation was assessed on the prognosis of leiomyomas in the oviduct of Japanese quail by providing 25 and 50 g of tomato powder for 365 days. Leiomyoma quantity was significantly reduced by tomato powder supplementation that was described by elevated serum lycopene, lutein, zeaxanthin, and vitamins A, C, and E, as well as by lowered MDA concentrations (Tinelli et al. 2021). Thompson and Cooney (2020) evaluated the relationship between plasma lycopene and β‐carotene levels in endometrial cancer cells. Serum lycopene and β‐carotenoids revealed a protective effect against endometrial cancer cells by elevating serum lycopene, β‐carotene, zeaxanthin, retinol, and tocopherol levels. An inverse correlation was observed between dietary fruit and vegetable consumption and uterine leiomyomata proliferation. Consumption of more than two servings of fruits and vegetables lowered the risk of uterine leiomyomata (Yang et al. 2022). A prospective study was conducted to investigate the lycopene effect against pre‐eclampsia and intrauterine growth retardation (IUGR) progression by supplementing two doses of lycopene (2 mg) to 135 primigravida women. It was observed that lycopene consumption lowered the incidence of pre‐eclampsia and IUGR in the affected women (Guerby et al. 2021).

### 1.16 Skin Cancer and Lycopene

Lycopene has effectively managed metabolic, cardiovascular, cancerous, reproductive, and inflammatory disorders (Shafe et al. 2024). Zhou et al. (2019) determined dietary lycopene's effects on chronic ultraviolet B‐induced skin carcinoma in SKH‐1 mice by feeding them a 1% lycopene diet for 2 weeks and 20 mJ/cm^2^ UVB irradiation thrice a week. The outcomes reduced pyrimidine dimer development and proliferative cellular nuclear antigen expression in irradiated skin. Lycopene inhibited onset, incidence, tumor multiplication, and tumor weight in affected mice. No significant variation was detected in protection between low or high lycopene concentrations. The protective effect of lycopene concentrations against acute UVB‐induced photodamage was studied, which concluded that lycopene inhibited MPO and ornithine decarboxylase, thereby reducing bifold skin thickness. Immunohistochemical staining showed an increased active apoptotic pathway of caspase‐3 in the affected group compared to the control group. Lycopene topically prevented caspase‐3 cleavage and reversed PCNA inhibition (Fazekas et al. 2003). Franco et al. (2012) conducted an observational study to evaluate dietary lycopene's potential in managing radiotherapy‐stimulated skin toxicity among 71 breast cancer patients. Two groups were devised: one group (*n* = 41) receiving topical prophylactic therapy, and another group of 30 patients was provided with lycopene (2 tablets/day). Results revealed more toxicity in control groups with PTV of more than 500 mL as compared to skin toxicity in the treated group. Another study was planned among Swiss albino mice to evaluate the anticancer properties of lycopene‐encapsulated nanoparticles. Nano‐lycopene exhibited potent antioxidant and anticancer effects against B16 cancerous cells. Moreover, nano‐lycopene reduced TPA‐induced skin edema, COX‐2 potential, oxidative stress, Bcl2, and Bax expression (Bano et al. 2020). The chemomodulatory potential of lycopene was studied against 9, 10‐dimethylbenz‐anthracene/12‐O‐tetradecanoylphorbol‐13‐acetate‐induced skin tumor and oxidative stress in female ICR mice. The results depicted lower tumor growth and proliferation by attenuating MDA and ROS, prohibiting glutathione loss, and attenuating oxidant enzyme activities in mice skin (Shen et al. 2014).

### 1.17 Thyroid Cancer and Lycopene

Thyroid cancer is a malignant form of cancer that is caused by oxidative stress stimulated by environmental pollutants, hazardous chemicals, and hormonal imbalance (dos Santos Valsecchi et al. 2024). An estimated 0.044 million cases of thyroid cancer were reported in the US, with approximately 2100 deaths from thyroid cancer. Al‐Amoudi (2018) evaluated the protective mechanism of lycopene on deltamethrin‐induced thyroid toxicity in albino rats. Lycopene administration inhibited DNA damage caused by deltamethrin and other cellular alterations such as hyperemia, epithelium lining, cytoplasm vacuolization, and rough endoplasmic reticulum expansion that develop during thyroid toxicity. Lycopene supplementation of 4 mg/kg/day for 30 consecutive days significantly reduced thyroid stimulating hormones (TSH) and MDA levels in Aroclor 1254‐induced cancer in the thyroid gland. Lycopene also suppressed inflammation, proliferation, and angiogenesis of thyroid cancer in rats (Ibrahim et al. 2021). Elsammak et al. (2023) evaluated lycopene's mitigating effect against tributyltin‐induced thyroid damage in adult male albino rats. The results revealed that lycopene treatment significantly improved caspase‐3 expression and thyroid structure. At the same time, lycopene treatment downregulated the expression of Beclin‐1, significantly ameliorating thyroid functioning. Lycopene supplementation reduces the oxidative stress caused by harness by enhancing the levels of SOD, catalase, and antioxidant capacity and improving the structures of thyroid tissues (Ismail et al. 2023). Lycopene acts synergistically with vitamin E to enhance thyroid hormone, thyroxine levels, and white ovarian follicles, consequently mitigating the risk of developing thyroid cancer (Ayo et al. 2022).

### 1.18 Lung Cancer and Lycopene

Lung cancer hurts the lungs of both genders and is associated with compromised lifestyle and socioeconomic status. Smoking and oxidative stress are the key parameters in the pathogenesis of lung cancer (Kratzer et al. 2024). The protective role of lycopene on lung cancer in males was studied by incorporating 25 and 50 ppm lycopene in drinking water for 21 weeks. The treatment revealed that 50 ppm of lycopene significantly reduced the proliferation of lung adenomas and lung carcinoma (Maddah et al. 2020). Cheng et al. (2020) investigated lycopene's potential in smoking‐accelerated oxidative stress in A549 by supplementing lycopene for 24 h. Lycopene masked lung cancer proliferation by suppressing oxidative stress as well as by elevating 8‐oxo guanine DNA glycosylase (OGG1), connexin‐43 (Cx43), and Nei‐like DNA glycosylases (NEIL1, NEIL2, and NEIL3) expression. Lycopene and anti‐PD1 combination effects were assessed by supplementing lycopene for lung cancer in mice. Lycopene and anti‐PD‐1 treatment synergistically decrease tumor weight, tumor volume, IL‐4, IL‐10, and PD‐L1 expression. On the other hand, cell apoptosis, IL‐1, IFN‐γ, and the CD4/CD8 ratio in the spleen of lung cancer‐suffering mice (Jiang et al. 2019).

**FIGURE 4:**
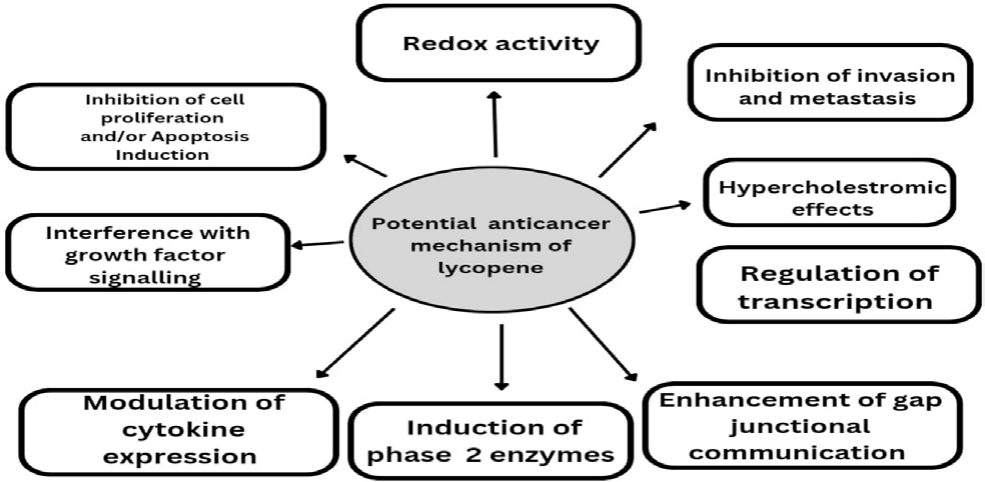
Lycopene mechanism of action for lung cancer prevention.

The conversion of lycopene to apo‐10‐lycopenoic acid had significantly reduced lung cancer progression by lowering cyclin E and cell cycle modulation from the G1 to S phase, as well as by activating p21 and p27 (cell cycle regulating proteins) in NHBE (normal human bronchial epithelial cells), BEAS‐2B, and A549 lung cancer cells (von Lintig et al. 2020). Mustra Rakic et al. (2019) investigated the preventive effect of lycopene against smoking‐induced chronic obstructive pulmonary disease (COPD). The supplementation of lycopene (~90 mg/kg/day) to ferrets for 22 weeks slowed down emphysema, chronic bronchitis, pre‐neoplastic lesions, and cholesterol accumulation. Lycopene elevated the expression of PPAR‐α, LXR‐α, and ATP‐binding cassette transporters (ABCA1 & ABCG1) in lungs, preventing the incidences of COPD in ferrets. The association between lycopene and sorafenib was studied against lung cancer progression. The results showed a positive association in the amelioration of lung cancer prognosis provoked by lowering the MAPK pathway and matrix metalloproteinase (MMP)‐2 and MMP‐9 (Chan et al. 2022). The lycopene mechanism of action for lung cancer prevention is shown in Figure 4.

### 1.19 Bladder Cancer and Lycopene

Bladder cancer affects the living standards of significantly large populations. Approximately 0.43 million individuals are diagnosed with bladder cancer each year (Xu et al. 2021). The protective mechanism of carotenoids for bladder cancer was evaluated among 242 bladder cancer patients, and it was observed that carotenoid‐rich products lowered cancer progression (Zhao et al. 2022). Kamal et al. (2022) and Wu et al. (2020) found an inverse correlation between carotenoids and bladder cancer pathogenesis. The severity and progression of bladder cancer were significantly reduced by the higher utilization of carotenoids in diet. A study investigated the link between fruit and vegetable consumption and bladder cancer progression. The results revealed that the uptake of cruciferous vegetables significantly reduced bladder cancer risk compared to fruit and vegetable intake (Kaiser et al. 2021). Table 1 shows the published data on the anticancerous potential of lycopene.

**TABLE 1:** Anticancerous potential of lycopene.

Cancer types	Investigation	Study type	Outcomes	Citations
Breast cancer	Investigating lycopene & β‐carotene's impact on MCF‐7, MDA‐235, and MDA‐231 cell lines	In vitro	Inhibition of cancerous cell propagation, cell growth, and apoptosis induction	Shin et al. (2020)
	Evaluation of lycopene on MCF, SK‐BR‐3, and MDA‐MB‐468 cell lines	In vitro	Suppression of cancer cell progression and cell cycle	Islam et al. (2022)
	Assessing the metastasis‐mitigating potential of lycopene against H‐Ras MCF10A and MDA‐MB‐231 cells	In vitro	Reducing H‐Ras MCF10A augmentation and development	Nalewajska et al. (2020)
Pancreatic cancer	Apoptosis‐inducing mechanism of lycopene for PANC‐1 cells	In vitro	ROS, NF‐kB, cIAP1, cIAP2, and survivin inactivation	Jeong et al. (2019)
	Evaluating lycopene impact on cerulein‐induced pancreatitis	In vitro	Decreased cytotoxicity, down‐regulating the NF‐kB pathway, JNK–caspase‐3 axis reduction	Agah et al. (2021)
	Determining the IL‐6 inhibition in pancreatic acinar cells	In vitro	Down‐regulating IL‐6 and NF‐kB expression, scavenging ROS species	Lee et al. (2022)
Prostate cancer	Exploring the repressive mechanism of lycopene for prostate hyperplasia	In vitro	PSA reduction, suppression of cancerous cell proliferation	Devlin et al. (2021)
	Investigating the prostate cancer‐suppressing effect of lycopene	In vitro	Enhancement of serum lycopene concentration, lowering cancer cell propagation	Ozkan et al. (2023)
	Inquiring about lycopene's potential in mitigating prostate cancer	In vitro	Improvement in serum lycopene concentrations, retardation of prostate cancer cell growth	Mia et al. (2023)
Colon cancer	Examining the lycopene inhibitory mechanism against HT‐29 cells	In vitro	Growth suppression, deactivation of the Akt signaling pathway, reduction of β‐catenin protein levels	Ağagündüz et al. (2022)
	Studying the preventive mechanism of lycopene and EPA on HT‐29 cancerous cells	In vitro	Inactivation of phosphatidylinositol 3‐kinase, Akt pathway down‐regulation, and Bax and Fas modulation	Kubczak et al. (2021)
	Analyzing the potential of lycopene against colorectal cancer	In vitro	Lowering PCNA and β‐catenin proteins, reduction of COX‐2, PGE_2_, and ERK1/2 proteins	Marino et al. (2023)
Oral cancer	Evaluating the cancer‐suppressing mechanism of lycopene against oral cancer	In vitro	Tumor cell proliferation and PI3K/AKT/m‐TOR signaling pathway inhibition	Wang et al. (2020)
	Assessment of lycopene on the KB‐1 cells	In vitro	Enhancing connexin‐43 and connexin‐43 expression	Abir et al. (2023)
	Evaluating lycopene and curcumin synergistic effect against OSCC	In vitro	Regression of cell viability & cell propagation	Girisa et al. (2021)
Liver cancer	Exploring the NAFLD‐mitigating potential of lycopene	In vitro	Reduction of body weight, serum LDL, TG, & TC, and activation of SOD and GPX	Kulawik et al. (2023)
	Inquiring about the NAFLD‐lowering activity of lycopene	In vitro	PPAR‐α, PPAR‐γ activation, hepatic inflammation & oxidative stress, reduction of CD_4_, CD_8_, IFN‐γ & TNF‐α, and macrophages	Yao and Liu (2022)
	Investigating lycopene's mitigating potential against HFD‐induced NAFLD	In vitro	SIRT1/AMPK & NAMPT pathway modulation and NF‐kB, IL‐6, IL‐1β, and TNF‐α suppression	Zhu et al. (2020)
Gastric cancer	Studying the anticancerous potential of lycopene against AGS, SGC‐7901, and Hs746T cell lines	In vitro	Suppression of mitochondrial membrane potential, apoptosis induction, and CCNE1, TP53, and MAPK pathway expression	Zhou et al. (2023)
	Evaluating the inhibitory potential of lycopene against oxidative stress injury‐induced gastric cancer	In vitro	Minimized oxidative DNA damage, HIF‐1α, Cyr61, NDRG1, BNIP3, and STAT2 mRNA, and enhanced SOD, CAT, & GPx activities	Cui et al. (2020)
	Exploring the gastric cancer‐lowering potential of lycopene	In vitro	Enhancement of IL‐4, IL‐10, Bax1, and caspase‐3; down‐regulation of IL‐6, TNF‐α, cyclin 1 & PCNA	Lim and Wang (2020)
Kidney cancer	Studying the ameliorating potential of lycopene against cisplatin‐induced nephropathy in male Wistar rats	In vitro	Body weight reduction and lowered levels of creatinine, urea, MDA, 8‐isoprostane, and renal Bax protein	Guo et al. (2021)
	Assessing the renal cell carcinoma protecting mechanism of lycopene	In vitro	Minimized tumor size, growth, length, and number, and limited tumor proliferation	Jurić et al. (2022)
	Inhibitory mechanism of lycopene in the progression of contrast‐induced nephropathy	In vitro	Elevated urea, creatinine, and MDA levels; reduced GSH, SOD, CAT, GPx levels; apoptosis stimulation	Tsigalou et al. (2020)
Brain cancer	Exploring the therapeutic potential of lycopene on hyperlipidemia‐induced brain injury	In vitro	Lowered levels of triglycerides, total cholesterol, LDL‐cholesterol, ox‐LDL, IL‐1, and TNF‐α, and improvement in LDL receptors, NGF, GABA, and 5‐HT	Yang et al. (2018)
	Investigating lycopene's potential in protecting against acrylamide‐induced neurotoxicity	In vitro	Enhanced levels of GSH, GPx, MDA, protein carbonyl, and neurotransmitters	Farouk et al. (2021)
	Assessing lycopene's potential in ameliorating β‐amyloid protein‐induced Alzheimer's disease	In vitro	Inhibited MDA levels, improved antioxidant capability	Guo et al. (2023)
Cervical cancer	Exploring the lycopene anticancerous mechanism in HeLa	In vitro	Improved expression of Bax & Nrf2, suppressed Bcl‐2 & NF‐kB pathway	Aktepe et al. (2021)
	Assessment of lycopene nanoparticles in cervical cancer management	In vitro	Lycopene silver nanoparticles revealed significant potential in lowering cervical cancer progression.	Shejawal et al. (2021)
	Inhibitory role of lycopene and carotenoids in HPV‐induced cervical cancer	In vitro	Suppression of HPV infection, elevated levels of serum lycopene and carotenoid concentrations	Ono et al. (2020)
Uterine cancer	Exploring the tomato powder effect on leiomyoma development in Japanese quail oviduct	In vitro	Improved lycopene, lutein, zeaxanthin, and vitamin A, C, and E levels, and limited MDA levels	Tinelli et al. (2021)
	Evaluating the lycopene relationship with endometrial cancer cells	In vitro	Enhanced serum lycopene, β‐carotene, retinol, and tocopherol levels downregulated the proliferation of cancerous cells	Thompson and Cooney (2020)
	Investigating lycopene's potential against the development of pre‐eclampsia and IUGR	In vitro	Reduced incidences of pre‐eclampsia and IUGR suffered by women	Guerby et al. (2021)
Thyroid cancer	Determining lycopene's potential in mitigating deltamethrin‐induced thyroid toxicity	In vitro	Lowered DNA damage, inhibited expansion of rough endoplasmic reticulum, and hyperemia	Al‐Amoudi (2018)
	Therapeutic potential of lycopene against Aroclor 1254‐induced cancer in thyroid cancer	In vitro	Lowered levels of TSH, MDA, inflammation, and growth of thyroid cancer	Ibrahim et al. (2021)
	Exposing the lycopene's protective effect on tributyltin‐induced thyroid damage in male albino rats	In vitro	Elevated levels of caspase‐3 expression, repair of thyroid structure, reduced levels of beclin‐1, and oxidative stress	Elsammak et al. (2023)
Lung cancer	Investigating lycopene's potential against lung cancer prevention	In vitro	Suppressed the proliferation of lung adenomas and lung carcinomas	Maddah et al. (2020)
	Exploring the lycopene effectiveness on smoking‐induced oxidative stress in A549 lung cells	In vitro	Masked lung cancer propagation, reduced oxidative stress, and up‐regulated the expression of connexin‐43, NEIL1, NEIL2, and NEIL3	Cheng et al. (2020)
Bladder cancer	Assessment of carotenoid mitigation potential of bladder cancer	In vitro	Elevating serum lycopene concentrations, lowering tumor growth	Zhao et al. (2022)
	Exploring the association of carotenoids in lowering bladder cancer incidences	In vitro	Inverse association was found between carotenoids and bladder cancer	Kamal et al. (2022)

## 2 Conclusions

Lycopene (red pigment), present in fruits and vegetables, particularly in tomatoes, offers promising therapeutic health attributes in managing and treating cancer owing to its cancer‐linked molecular pathways suppressive mechanism. The absorption and bioavailability of lycopene are improved by incorporating fatty foods into the diet. Studies reveal that lycopene can hamper DNA damage, oxidative stress, and inflammation, thus slowing down the progression of cardiovascular and cancerous disorders. The wide range of attributes associated with lycopene is crucial in the pharmaceutical and nutraceutical industries and in the production of functional foods, making it a critical compound for nurturing well‐being and overall health. Its effects are notable in colon, breast, prostate, pancreatic, and oral cancers. The compound has been observed to induce apoptosis, hinder tumor growth, and diminish oxidative stress by regulating primary molecular pathways, which include mTOR/AKT/PI3K, NF‐kB, and Wnt signaling. Its time‐ and dose‐dependent efficacy in the prevention and hindering of the proliferation of cancer cells has been documented by several studies. Overall, the anticancer potential of lycopene offers a promising platform for developing therapeutic and preventive strategies in the field of oncology.

## Author Contributions

**Muhammad Maaz:** conceptualization (equal), writing – original draft (equal). **Muhammad Tauseef Sultan:** conceptualization (equal), writing – original draft (equal). **Muhammad Usman Khalid:** data curation (equal), investigation (equal). **Hassan Raza:** writing – review and editing (equal). **Muhammad Imran:** resources (equal), validation (equal), visualization (equal). **Muzzamal Hussain:** supervision (equal), writing – review and editing (equal). **Waleed Al Abdulmonem:** investigation (equal), methodology (equal). **Suliman A. Alsagaby:** writing – review and editing (equal). **Mohamed A. Abdelgawad:** data curation (equal), validation (equal), visualization (equal). **Mohammed M. Ghoneim:** data curation (equal), investigation (equal). **Muhammad Asif Khan:** writing – review and editing (equal). **Tadesse Fenta Yehuala:** formal analysis (equal), supervision (equal), visualization (equal), writing – review and editing (equal). **Samy Selim:** writing – review and editing (equal). **Ehab M. Mostafa:** data curation (equal), investigation (equal), resources (equal).

## Conflicts of Interest

The authors declare no conflicts of interest.

## Data Availability

The data that support the findings of this study are available on request from the corresponding author.
